# Diabetes conversation map - a novel tool for diabetes management self-efficacy among type 2 diabetes patients in Pakistan: a randomized controlled trial

**DOI:** 10.1186/s12902-020-00572-x

**Published:** 2020-06-16

**Authors:** Rubina Qasim, Sarfaraz Masih, Mohammad Tahir Yousafzai, Hakim shah, Abdul Manan, Yousaf Shah, Muhammad Yaqoob, Abida Razzaq, Ajmal Khan, Atiya Rahman Khan Rohilla

**Affiliations:** 1 Dow University of Health Sciences, Karachi, Pakistan; 2 Indus college of Allied Health, The Indus Hospital, Karachi, Pakistan; 3 Department of Pediatrics and Child Health, Aga Khan University, Karachi, Pakistan; 4 Patel Hospital and Institute of Nursing, Karachi, Pakistan; 5 Liaquat National Hospital College of Nursing, Karachi, Pakistan

**Keywords:** Diabetes management, DMSE, Diabetes mellitus type 2, Conversation map, Diabetes education, Diabetes management self-efficacy, Pakistan, Lower middle-income countries

## Abstract

**Background:**

This study aimed to measure the effect of diabetes education using the novel method of “diabetes conversation map (DCM)” as compared to routine counselling (RC) on diabetes management self-efficacy (DMSE) among patients living with type 2 diabetes in Karachi, Pakistan.

**Methods:**

A parallel arm randomized controlled trial among patients with type 2 diabetes aged 30–60 years, with HbA1c > 7%, diagnosed for at least 5 yrs., was conducted at the national institute of diabetes and endocrinology in Karachi, Pakistan. A total 123 type 2 diabetes patients were randomized into DCM (*n* = 62) or RC (*n* = 61). Four weekly diabetes control sessions of 40 min each using the DCM or RC was provided. DMSE was measured using a validated Urdu language DMSE tool at baseline and after three months of the randomization. Change in DMSE and HbA1c levels within groups (pre-post) and between the groups after 3 months of enrollment was compared.

**Results:**

Baseline characteristics except HbA1c were similar between the two arms. After 3 months of enrollment, there was no change in the DMSE score in the RC arm however, significant increase in DMSE score was noted in the DCM arm (*P* = < 0.001). The average difference (95% confidence interval) in DMSE score between the DCM and RC arm was 33.7(27.3, 40.0; *p* = < 0.001) after 3 months of the enrollment. Difference in HbA1c within groups was not significant.

**Conclusions:**

DCM significantly improved DMSE among type 2 diabetes patients in a developing country setting like Pakistan. Healthcare workers caring for type 2 diabetes patients need to be trained on DCM to effectively utilize this novel tool for educating diabetes patients.

**Trial registration:**

This trial was prospectively registered. ClinicalTrials.gov Identifier: NCT03747471. Date of registration: Nov 20. 2018.

## Background

The world has observed more than four times increase in the number of cases of adult diabetes during the last four decades. The number of adult diabetes cases increased from 108 million in 1980 to 463 million in 2019 and the projections for 2045 is about 700 million [1, 2]. Globally, about a third of the diabetes burden is in the low middle income countries (LMIC). Pakistan being a LMIC, also experienced an increase from 1.7 million of adult diabetes patients in 1980 to 19.4 million in 2019 and is considered as one of the top 10 countries with highest number of adult population living with diabetes (diabetes prevalence of ≥12%).(1; 2) In 2019, about 4.2 million deaths attributed to diabetes and diabetes related complications occurred globally and almost half (46.2%) of the deaths (majority from lower middle income countries) were among the working age group (< 60 years old) [2, 3]. According to the sustainable development goals (SDG), by 2030 the premature deaths attributed to non-communicable diseases should be reduced by 1/3rd globally [4]. Since the life style habits including daily routine are associated with diabetes and its control, therefore people living with diabetes can play an important role in their disease management [5].

### Diabetes management self-efficacy (DMSE)

Self-efficacy (SE) is considered as an essential pre-requisite for the initiation and adoption of healthy lifestyle habits [6, 7]. In 1977, Albert Bandura described SE as one’s ability to influence or perform actions that affects life and to exercise control over these actions. Also, Bandura proposed that people take actions when they believe they can do it and hesitate to take actions when they do not believe they can be able to perform [8]. Several studies have reported the association between self-efficacy and diabetes management, blood glucose levels, quality of life and eating behaviors among people living with diabetes [9–12]. Thus, self-efficacy is considered as an important factor which can play an important role in the management of diabetes mellitus.

### Diabetic conversation map (DCM)

The orthodox methods of teaching people with diabetes to control their disease, prevent complications, and improve quality of life, are predominantly through didactic lectures, brochures, pamphlets, and routine counselling [13, 14]. While these methods have shown some benefit in terms of diabetes control among patients, studies have reported level of education among patients as one of the important predictor for these benefits [15–17]. Among novel methods, Diabetes Conversation Maps (DCM) are considered as useful tools for the education of diabetes patients [18]. DCM are interactive tools containing pictorial messages and directions designed to educate patients with diabetes and their family members. Since the messages are predominantly self-explanatory depicted in pictures, it does not need formal education. In LMIC like Pakistan with a less than 50% literacy rate, these tools might prove to be effective for the control of diabetes and its associated complications [19].

DCM have already been tested in several countries across the world [20, 21]. In the developing world, it has shown some impact through observational studies however, literature generated from randomized control trials is limited [22, 23]. Factors such as formal education, access to the modern modes of communication (internet, social media), sociodemographic characteristics and environmental exposures are different in the LMIC as compared to the first world countries and hence there is a need to test the effectiveness of DCM in the developing world context. Therefore, this study aimed to measure the effectiveness of DCM versus routine counselling (RC) to improve DMSE among patients living with type 2 diabetes in LMIC setting like Karachi, Pakistan.

## Methods

Detail methodology of the study is available in the published study protocol [24].

### Design, setting and randomization

This was two 1:1 parallel arm unblinded randomized controlled trial conducted during November 26, 2018 to May 30, 2019. The study was conducted at National Institute of Diabetes and Endocrinology (NIDE), Dow University of Health Sciences, Karachi, Pakistan. NIDE is one of the largest public sector diabetes centers located in the heart of the metropolitan city of Karachi, Pakistan. The center provides outpatient, inpatient, and diagnostic facilities at subsidized rates to all diabetes patients of Karachi and other parts of Sindh province (Southern Pakistan). The daily turnover (Monday to Saturday) in the outpatient department is more than 200 diabetes patients. A unique computerized medical record number is provided to every patient visiting the hospital for the first time. All the medical records including record of laboratory investigations being performed at the hospital are archived using the unique medical record number.

The individual randomization was performed by the one of the investigators (MTY) through the generation of random digit numbers from 1 to 120 using Microsoft excel 10 (RANDBETWEEN function). Random digit numbers ≤60 was assigned to the DCM arm and numbers above 60 were assigned to the RC arm. Random digit numbers with corresponding assignments were sealed in the opaque envelops by MTY, kept in sequence and the other investigator (RQ) opened the envelops consecutively as patients were screened and found eligible for enrollment into the study.

### Population and sample size

The inclusion criteria for enrollment of patients was aged 30–60 years, visiting outpatient department of NIDE, already diagnosed with type 2 diabetes for ≥5 years, HbA1c levels> 7%, and positive for diabetes distress (DD) using the validated DD screening tool [25]. Patients with type 2 diabetes suffering from major disabilities, mental illness, severe complications related to diabetes, and living outside Karachi were excluded from this study.

The sample size for this trial was calculated for two different objectives. Objective 1 related to the impact of DCM on DMSE and objective 2 related to the impact of DCM on DD. While the sample size calculation for both the objectives is provided here, data from objective 2 is not included in this manuscript. The sample size for objective 1 was estimated based on the findings from a randomized controlled trial conducted among patients with type 2 diabetes in rural Thailand [26]. The study tested the effectiveness of family oriented diabetes self-management education versus routine care to improve diabetes management self-efficacy (DMSE) using the validated DMSE scale [27]. The study reported average ± standard deviation DMSE scores of 55.6 ± 12 at baseline which increased to 76 ± 9.4 after 13 weeks of enrollment in the intervention arm and 58.7 ± 11.4 which increased to 60.7 ± 13.1 in the control arm. DMSE scores at baseline were similar between the two groups, however at 13 weeks the difference between the two scores was highly significant (*p* = < 0.001). Considering the difference of 15 in the average DMSE scores [26] between the DCM and RC after 3 months of randomization, assuming 95% confidence level, 90% power to detect the given difference in two arms, adjustment for 10% attrition rate, the minimum sample size for objective 1 was *N* = 40 (20 patients in each arm). For objective 2, the sample size was estimated based on a pilot randomized controlled trial conducted in China [28]. The study reported average ± standard deviation score of DD (DD measured through a validated screening tool [25]) between control and intervention group at baseline as 32.77 ± 14.57 and 26.08 ± 9.92 respectively (*p* value = 0.073). After 6 months of the intervention the respective scores were 30.09 ± 12.14 and 22.79 ± 4.95 (p value = 0.014) between the two groups, respectively. Considering a higher average difference of the differences (difference of 7.30) as compared to the study from china (difference of 0.62) [28] between the two arms (which is also clinically significant), assuming 95% confidence level and 80% power to detect the given difference, the minimum sample size to achieve the objective 2 was *N* = 88 (44 in each group). After adding attrition rate of 30%, a total of 120 participants (60 in each arm) were needed. Since the sample size for objective 2 was higher than the sample size for objective 1, hence it was used for enrollment in this study.

### Operational definitions

Diabetes control: Patients with type 2 diabetes with glycated hemoglobin A1c (HbA1c) ≤7% were considered with a controlled diabetes.

Suboptimal control of diabetes: Patients with type 2 diabetes with HbA1c more than 7% were considered with suboptimal control of diabetes.

### Enrollment and data collection procedure

Detailed enrollment and data collection procedure is provided in the published protocol of this study [24]. After necessary screening for eligibility and seeking written informed consent for participation, eligible participants were enrolled and randomized into the two arms. We used a structured pilot tested questionnaire developed in English, translated into Urdu and back translated to English for the data collection. Data was collected through face-to-face interview by the Principal Investigator (RQ) of this project. Clinical, laboratory and latest anthropometric data such as treatment, comorbidities, any complications, HbA1c, any other laboratory tests being performed recently, height and weight were retrieved from the respective medical record file. The same questionnaire was used for the follow-up data collection after 3 months of the randomization. English version of the questionnaire is already published [24].

The DMSE was measured using validated Urdu version of the DMSE scale [27]. The scale has 20 Likert items, each with response ranging from 0 to 10 (0 = completely unable, 10 = completely able). DMSE scale total possible score ranges from 0 to 200, with higher score representing higher diabetes management self-efficacy. Internal consistency of the scale based on data from this study was satisfactory (Cronbach’s alpha = 0.96). Factor analysis of the DMSE scale based on our data revealed four different dimensions measured by the scale; 1) exercise and weight control, 2) diabetic treatment, 3) blood glucose monitoring, and 4) diet control. There were 10 Likert items in the DMSE sub-scale related to exercise and weight control with the score range 0–100. In the sub-scale related to diabetes treatment, there were 5 Likert items with score range from 0 to 50. Similarly, in the sub-scales related to blood glucose monitoring and diet control, there were 3 and 2 Likert items respectively with the score range from 0 to 30 and 0–20 respectively. Further details on validation and reliability assessment of the DMSE scale are beyond the scope of this paper and will be published later.

### Intervention and follow-ups

Participants in the DCM arm received 4 education sessions each (in groups of 6–8 participants) of 40 min duration using the standard pictorial colorful conversation maps [18]. The pictorial maps addressed issues related to managing diabetes, healthy lifestyle, starting insulin, and experiencing life with diabetes. There were four different DCM tools, each with a specific domain related to diabetes self-management such as 1) living with diabetes, 2) how diabetes works, 3) healthy eating and keeping active, and 4) starting insulin. (Additional file 1, DCM maps adopted from healthy interactions catalog and translated in Urdu by Lilly pharmaceuticals Pvt. Ltd. Pakistan. Permission for use and publication obtained from Lilly Pakistan Ltd. which is a partner of Healthy Interactions LLC 311 W. Superior St. Chicago, IL USA.).

Participants in the RC attended 4 routine diabetes counseling provided by the diabetes clinics’ trained nurses. The counselling sessions were provided in groups of 6–8 and each session ranged from 30 to 40 min. The sessions in both DCM and RC arms were organized on weekly basis, started immediately after randomization. (Table 1) Separate counselling room located in the same building of the consultant clinics was used for teaching the participants in the DCM arm while the participants in RC arm received counselling as usual in the outpatient department area.

**Table 1:**
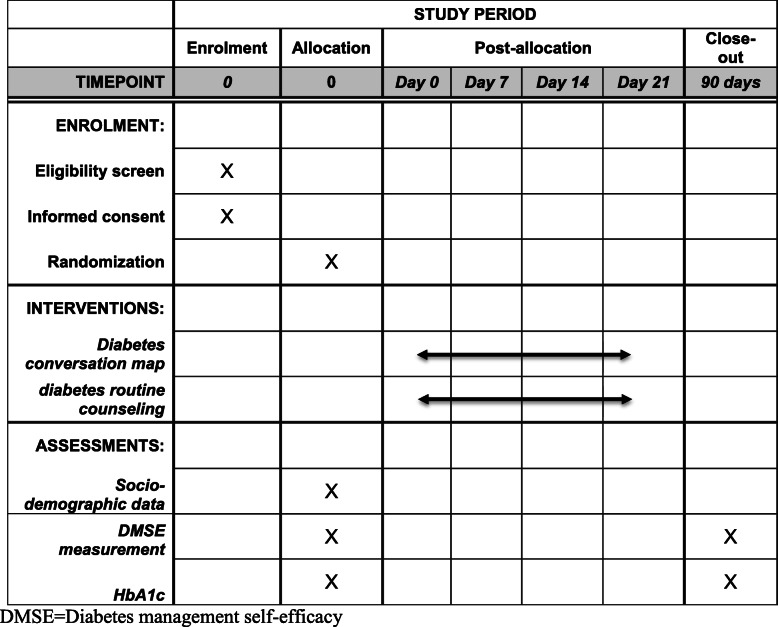
Checklist for the schedule of enrolment, interventions, and assessments

*DMSE* Diabetes management self-efficacy

### Statistical analysis

Data was entered into statistical package for social scientists (SPSS) version 25 (IBM®). Intention to treat analysis was performed. Data cleaned and any discrepancies or missing information were validated against the physical questionnaires and/or medical record files. Body mass index (BMI) was computed using the formula weight in kilogram/(height in meters)^2^ and classified into three categories based on recommendations of WHO expert consultation for BMI categories for Asian population for public health action. BMI 18.5 to < 23 was categorized as acceptable risk, 23 to 27.5 as increased risk and ≥ 27.5 as high risk [29]. Variables containing sum of scores for DMSE, and its sub-scales (factors) were computed by adding the respective Likert scale items. Assumptions for parametric tests especially normality and homogeneity of variance were tested for all continuous scale variables. Descriptive analysis was performed by using frequency with percentages for all categorical variables and mean with standard deviations for all continuous scale variables. The distribution of all variables including independent (demographic, anthropometric, lifestyle habits and clinical characteristics) and dependent (DMSE and its sub-scales) were compared at baseline between the intervention (DCM) and control (RC) arms. *P* values were calculated using Pearson Chi Square test for categorical and independent sample T test for the continuous scale variables. Data on DMSE, DMSE sub-scales and HbA1c at 3 months following the randomization were compared using the independent sample T test. Differences with *P* values of less than 0.05 were considered significant.

## Results

**Fig. 1:**
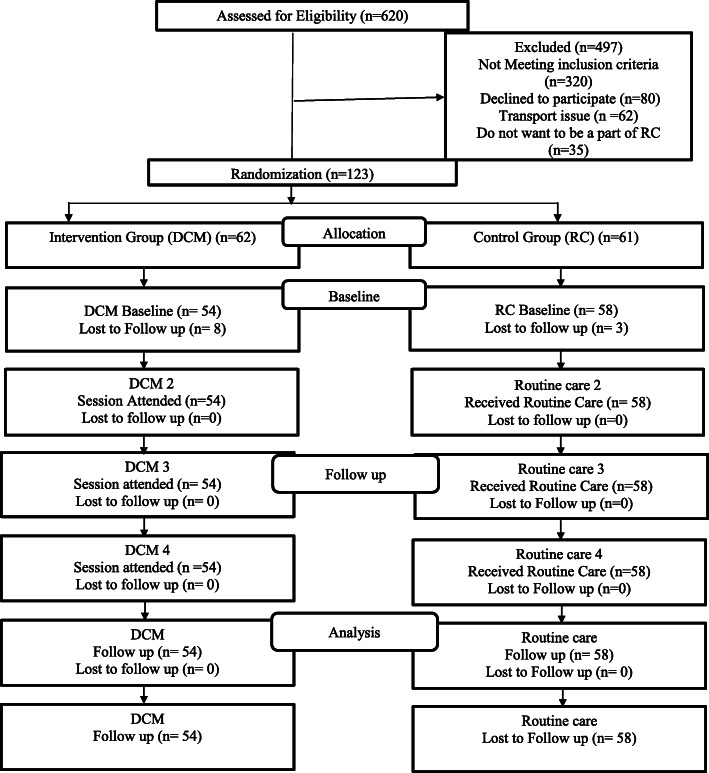
Consort diagram showing the number of participants at enrollment and follow-ups

We approached 620 type 2 diabetes patients and 123/620(19.8%) were eligible for enrollment. Out of 123 participants, 61(49.6%) were randomized into the RC and 62(50.4%) were randomized into the DCM arms. Among total randomized into the two arms, only 58/61(95%) received routine counselling and also provided baseline data in the RC arm and only 54/62(87%) received education based on conversation map and provided baseline data in the DCM arm. There was *n* = 3 and *n* = 8 patients in the RC and DCM arm respectively who withdrew their consent after randomization and did not provide baseline data. There was no loss to follow-up in the subsequent four visits. Figure 1 is a consort diagram showing the flow of the patients in both arms.

At baseline, there was no significant difference in terms of age, gender, monthly income, level of education, and marital status between the two arms. Overall, about 75% of the participants were 45–60 years old. About half of the participants were female with a slightly higher proportion in the DCM arm (57%) as compared to the RC arm (45%) however the difference was not statistically significant (*p* value 0.183). Similarly, at baseline, lifestyle habits e.g. activity pattern, smoking status, substance abuse and body mass index (BMI) were similar between the two arms. Overall, slightly less than half (40%) of the participants in this study were high risk (BMI > 27.5). (Table 2).

**Table 2:** Comparison of baseline sociodemographic, lifestyle and anthropometric data the two arms (*N* = 112)

	Total	DCM	RC	
**Sociodemographic**	***N*** **= 112(%)**	***n*** **= 54(%)**	***n*** **= 58(%)**	***P*value**
**Age groups (Yrs)**				
30 - < 45	28 (25)	14 (25.9)	14 (24.1)	0.827
45–60	84 (75.0)	40 (74.1)	44 (75.9)	
**Gender**				
Male	55 (49.1)	23 (42.6)	32 (55.2)	0.183
Female	57 (50.9)	31 (57.4)	26 (44.8)	
**Monthly income (PKR)**				
≤ 25,000	20 (17.9)	8 (14.8)	12 (20.7)	0.704
26,000–50,000	41 (36.6)	20 (37.0)	21 (36.2)	
> 50,000	51 (45.5)	26 (48.1)	25 (43.1)	
**Education level**				
≤ Secondary	65 (58.0)	27 (50.0)	38 (65.5)	0.096
> Secondary	47 (42.0)	27 (50.0)	20 (34.5)	
**Marital status**				
Single/widow/divorce	4 (3.6)	4 (7.4)	0	0.051
Married	108 (96.4)	50 (92.6)	58 (100)	
**Activity pattern**				
Active	77 (68.8)	36 (66.7)	41 (70.7)	0.646
Sedentary	35 (31.3)	18 (33.3)	17 (29.3)	
**Smoking**				
Yes	9 (8.0)	3 (5.6)	6 (10.3)	0.492
No	103 (92.0)	51 (94.4)	52 (89.7)	
**Substance abuse**				
Yes	11 (9.8)	4 (7.4)	7 (12.1)	0.407
No	101 (90.2)	50 (92.6)	51 (87.9)	
**BMI (kg/m2)** based on cut-off points for public health action in Asian population				
(Increasing but acceptable risk) 18.5- < 23	24 (21.8)	15 (28.3)	9 (15.8)	0.275
(Increased risk) 23–27.5	42 (38.2)	18 (34.0)	24 (42.1)	
(High risk) > 27.5	44 (40.0)	20 (37.7)	24 (42.1)	

*DCM* Diabetes Conversation Map (Intervention arm), *RC* Routine care (Control arm)

*P* values are two sided calculated based on Pearson chi-square or fisher exact test for the categorical variables and independent samples t test for the continuous variables

At baseline, average score of DMSE and DMSE sub-scales, history of diabetes, and co-morbidities were similar between the two arms. However, participants in the RC arm had average HbA1C level of 10 ± 1.8 as compared to 9.0 ± 1.5 in the DCM arm (*p* value = 0.042). Similarly, current diabetes treatment was statistically different between the two arms. About a quarter (28%) of the participants in the DCM arm were receiving insulin as compared to 16% of the participants in RC arm and 67% of the participants receiving oral hypoglycemic drugs in the RC arm as compared to 43% in DCM arm (*P* value 0.032). (Table 3).

**Table 3:** Comparison of baseline clinical characteristics, DMSE and sub-factors of DMSE between intervention and control arms (*N* = 112)

	Total	DCM	RC	
**Clinical characteristics**	***N*** **= 112(%)**	***n*** **= 54(%)**	***n*** **= 58(%)**	***P*value**
**Family history of diabetes**				
Yes	85 (75.9)	41 (75.9)	44 (75.9)	0.994
No	27 (24.1)	13 (24.1)	14 (24.1)	
Duration of diagnosis of diabetes (Mean ± SD yrs)	9.5 ± 5.8	10.2 ± 6.3	8.8 ± 5.3	0.209
HbA1c at baseline (Mean ± SD)	9.3 ± 1.7	9.0 ± 1.5	10.0 ± 1.8	0.042
**Comorbidities**				
Hypertension	48 (92.3)	25 (92.6)	23 (92.0)	0.936
Other (CHD, COPD)	4 (7.7)	2 (7.4)	2 (8.0)	
No information	–	–		
**Treatment**				
Insulin	24 (21.4)	15 (27.8)	9 (15.5)	0.032
Oral	62 (55.4)	23 (42.6)	39 (67.2)	
both	26 (23.2)	16 (29.6)	10 (17.9)	
Ovrall DMSE at baseline (Mean ± SD)	81.7 ± 17.2	81.6 ± 16.9	81.8 ± 17.4	0.938
Factor 1:DMSE exercise & weight control (Mean ± SD)	36.5 ± 10.3	35.3 ± 9.8	37.6 ± 10.8	0.241
Factor 2: Health seeking and diabetes treatment (Mean ± SD)	29.5 ± 7.1	30.6 ± 7.3	28.4 ± 6.8	0.112
Factor 3:Blood sugar monitoring (Mean ± SD)	11.2 ± 2.7	11.1 ± 2.7	11.2 ± 2.7	0.72
Factor 4:Diet control (Mean ± SD)	4.5 ± 1.9	4.6 ± 2.0	4.5 ± 1.7	0.798

*DCM* Diabetes Conversation Map (Intervention arm), *RC* Routine care (Control arm)

*P* values are two sided calculated based on Pearson chi-square or fisher exact test for the categorical variables and independent samples t test for the continuous variables

While there was no statistically significant difference in DMSE score at the baseline, at 3 months follow-up the average difference in DMSE score (95% confidence interval) increased to 33.7(27.3, 40.0) between the DCM and RC arm (*p* value = < 0.001). Besides, there was no significant difference in average scores of the DMSE sub-scales between the two arms at the baseline however, after 3 months the average score of DMSE in DCM arm increased significantly as compared to the RC arm. While the difference in average HbA1c level between the DCM and RC arms was statistically significant at both baseline and 3 months follow up, the reduction in HbA1c within the groups (pre-post) in both the DCM and RC arms was not statistically significant to achieve diabetes control (HbA1c < 7%) in any of the two groups. This should be noted that treatment regimen remained same during the study period in both the groups while 1 unit decline in HbA1c was noticed in both RC and DCM arm. (Table 4).

**Table 4:** Comparison of diabetes management self-efficacy, and HbA1c after 3 months of enrolment (*N* = 112)

Clinical characteristics	DCM	RC	Difference(95%CI)	***P*** value
Overall DMSE score after 3 months of enrolment (Mean ± SD)	115.5 ± 18.0	81.9 ± 15.8	33.7 (27.3,40.0)	< 0.001
Factor 1:Exercise & weight control (Mean ± SD)	56.2 ± 10.5	37.1 ± 9.1	19.0 (15.3,22.7)	< 0.001
Factor 2: Health seeking and diabetes treatment (Mean ± SD)	36.1 ± 4.9	28.3 ± 5.6	7.9 (5.8,9.8)	< 0.001
Factor 3:Blood sugar monitoring (Mean ± SD)	16.6 ± 3.1	11.7 ± 2.8	4.8 (3.7,5.9)	< 0.001
Factor 4:Diet control (Mean ± SD)	6.6 ± 2.3	4.6 ± 2.2	1.9 (1.1,2.8)	< 0.001
HbA1c post intervention (Mean ± SD)	8.2 ± 1.4	9.1 ± 1.8	−0.92(−1.68, −0.16)	0.025

*DCM* Diabetes Conversation Map (Intervention arm), *RC* Routine care (Control arm)

*P* values are two sided calculated based on independent sample t test between intervention and control arm

## Discussion

This study is first in the region evaluating the effectiveness of DCM against RC to improve DMSE. In this study four education sessions on diabetes using DCM as compared to RC significantly improved the DMSE score. Similarly, this study also observed significant improvement in the DMSE sub scales (Exercise and weight control, health seeking and diabetes treatment, blood sugar monitoring, and diet control) in the DCM arm as compared to the RC arm. In this study, significant difference was observed in HbA1c between DCM and RC arms however, the difference in HbA1c levels within each arm (post-pre) was not statistically significant.

We could not find any other study evaluating the effect of DCM on DMSE however, several studies from different countries including Pakistan has assessed the impact of DCM on other outcomes among patients with diabetes mellitus. A randomized controlled pilot trial in China studied the effect of DCM as compared to traditional education on diabetes distress and diabetes empowerment among patients with type 2 diabetes mellitus. They observed significant increase in the diabetes empowerment score at 6 months following enrollment in the intervention arm as compared to the control [28]. While the study in China reported the effectiveness of DCM to improve empowerment among diabetes patients, their outcome (diabetes empowerment) was not similar to our outcome (DMSE) and hence cannot be compared. Besides, the sample size in Chinese study was only 53 as compared to our study where the number of participants was more than doubled. Similarly, in Chinese study the last follow up was 6 months after the enrollment as compared to our study where the last follow up for measurement was only 3 months after the enrollment. Another randomized controlled trial conducted among patients with type 2 diabetes mellitus found significant impact of DCM as compared to the routine care on patients’ satisfaction with care, perception of goal attainment and knowledge of diabetes after 6 months of the intervention [30].

A cross sectional study conducted at a tertiary care hospital in Karachi Pakistan where diabetic education was provided through DCM found significant improvement in knowledge, attitude and practices of the patients with type 2 diabetes mellitus [23]. Unlike these studies, a recently conducted systematic review which aimed to generate evidence regarding the effect of DCM on the behavior related to drug adherence, level of HbA1c and blood pressure control, reported inconclusive evidence. The authors of the systematic review concluded that DCM has the potential to positively affect the behavior and health outcome of type 2 diabetes patients however, sufficiently powered, well designed studies are recommended to fill the existing knowledge gap [31].

In this study, we could not find the significant impact of DCM on HbA1c, however 1unit decline in HbA1c was noted in both RC and DCM arms as compared to the baseline HbA1c while the antidiabetic treatment was not changed during the study duration. This finding is consistent with another study from Spain where DCM as compared to routine care was found to be significantly associated with improvement in knowledge however, no impact on clinical outcomes including HbA1c [30]. There could be several reasons for the lack of association between DCM and improvement in HbA1c. Firstly, HbA1c is an indicator of long term glycemic control indicating the cumulative glycemic history for the last 2–3 months [32]. Since, we measured the change in HbA1c only after 3 months of the enrollment and during the initial 1 month, 4 weekly sessions were conducted thus the time to bring change in the daily routines, behaviors, practices based on the education resulting in improvement of HbA1c might not be sufficient. Secondly, studies in the past have reported that improvement in the HbA1c level is relatively difficult among diabetes patients with longer duration of the illness as compared to the patients who are newly diagnosed [33]. Since the average of duration of illness in this study was 10 and 9 years among patients in the DCM and RC arms respectively, therefore, longer duration might have resulted in no significant improvement in HbA1c during the 3 months duration after enrollment. Future studies with longer duration of follow-up after the intervention are recommended to evaluate the impact of DCM on HbA1c.

In this study, we also found significant improvement in the scores based on DMSE sub-scales for example, 1) exercise and weight control, 2) health seeking and diabetes treatment, 3) blood sugar monitoring, and 4) diet control. This has important implications for decreasing the treatment non-compliance, lack of responsibility for self-care, dietary control, health seeking behavior and weight management [21].

Important limitations should be considered before drawing any conclusion based on this study. First, this trial was conducted only in one public sector tertiary care hospital in Karachi and generalizability to other settings might be limited. Second, our follow-up of 3 months after enrollment might not be sufficient to measure a change in the clinical outcome e.g. HbA1c. However, these patients are regularly visiting the given hospital and their HbA1c levels are routinely measured. The HbA1c data on 6 months and 9 months following recruitment in the study can be retrieved subject to the approval of the revised ethical application. On the other hand, one of the important strengths of this study is 100% retention of the participants during the follow-up period. The higher retention was possible due to excellent rapport between the study participants and the study staff, phone call reminders at least 1 day before the scheduled visit and better time management during the sessions (no waiting time and finishing sessions on time). Besides, validated tools in local language for the assessment of DMSE was used.

## Conclusions

DCM performed better as compared to the routine care in improving DMSE among patients with type 2 diabetes in a developing country like Pakistan. Clinicians and nurses dealing with diabetic patients are recommended to use DCM rather than routine counselling methods for the education of their patients especially in developing countries.

## Supplementary information

**Additional file 1.**

## Data Availability

The datasets used and/or analyzed during the current study are available from the corresponding author on reasonable request.

## Abbreviations

DCM: Diabetes conversation map
DMSE: Diabetes management self-efficacy
DD: Diabetes distress
NIDE: National institute of diabetes and endocrinology
Type 2 DM: Type 2 diabetes mellitus
DUHS: Dow university of health sciences
IRB: Institutional review board
RC: Routine care
LMIC: Low middle-income country
BMI: Body mass index
