# Supplementary figures and images for: Diabetes conversation map - a novel tool for diabetes management self-efficacy among type 2 diabetes patients in Pakistan: a randomized controlled trial
Source: BMC Endocr Disord. 2020 Jun 16;20:88. doi: 10.1186/s12902-020-00572-x (PMC7298747; doi:10.1186/s12902-020-00572-x)

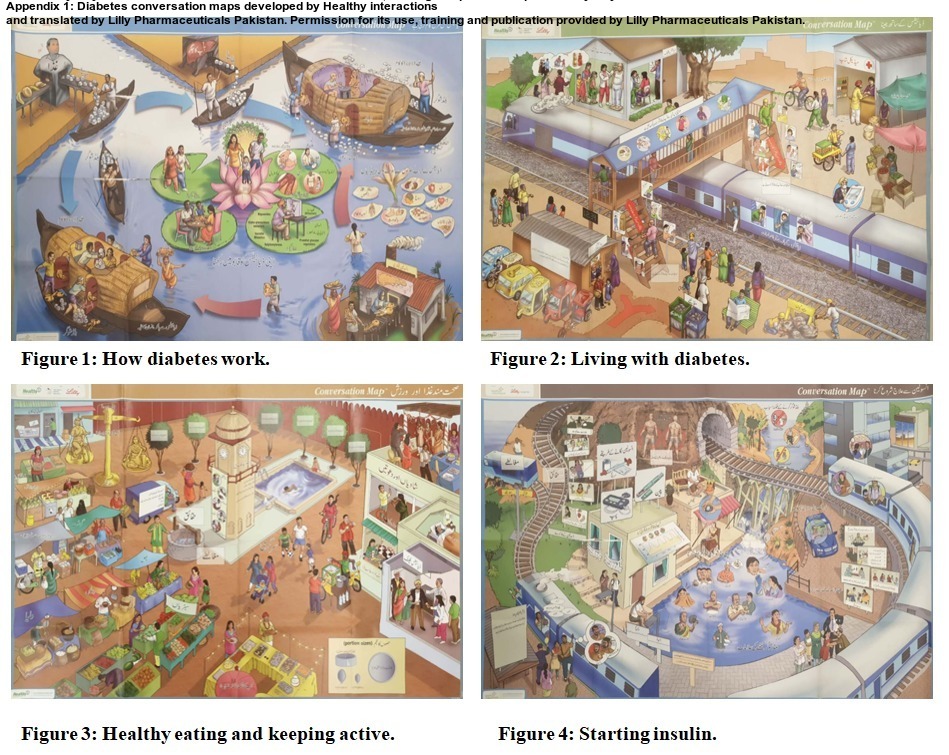

Supplement: Supplementary file 1 — Additional file 1. [file 12902_2020_572_MOESM1_ESM.jpg]
